# Trend of antibiotic susceptibility of *Streptococcus pyogenes* isolated from respiratory tract infections in tertiary care hospital in south Karnataka

**Published:** 2019-02

**Authors:** Anupam Berwal, Kiran Chawla, Seema Shetty, Ashu Gupta

**Affiliations:** 1 Department of Microbiology, Kasturba Medical College, Manipal Academy of Higher Education, Manipal, India; 2 Department of Microbiology, Deendayalupadhyay Hospital, New Delhi, India

**Keywords:** *Streptococcus pyogenes*, Penicillin, Resistance, Minimum inhibitory concentration

## Abstract

**Background and Objectives:**

*Streptococcus pyogenes* is recognized as an important pathogen of respiratory tract infections. The rapidly, emerging problem of antibiotic resistant *Streptococcus pyogenes* is a major issue nowadays. The present study aimed to evaluate the antibiotic susceptibility of *Streptococcus pyogenes* isolated from upper respiratory tract infections in tertiary care hospital of south Karnataka.

**Materials and Methods:**

A retrospective study was conducted over a period of two years. The specimens were processed by Gram staining and aerobic culture. The bacteria were isolated as per standard protocols. The minimum inhibitory values and extent of antibiotic resistance of commonly used antimicrobials were analysed for the isolated strains.

**Results:**

A total of 2123 specimens were received from patients with respiratory tract infections, among which, 50 *Streptococcus pyogenes* isolates were obtained. Out of these, 8% were not sensitive to penicillin. Using VITEK 2 system, the prevalence of resistances to cefotaxime, erythromycin, tetracycline, levofloxacin, clindamycin and ceftriaxone were 4.2%, 83%, 51%, 8.9%, 40% and 5.3% respectively.

**Conclusion:**

It is important to know about the prevalence of resistance and rising MIC values of commonly used antibiotics regarding *Streptococcus pyogenes* to avoid therapeutic failures.

## INTRODUCTION

Group A streptococcus (GAS), or *Streptococcus pyogenes*, is a facultative, Gram-positive β-hemolytic cocci which causes wide range of diseases in humans, from mild to life-threatening ones, such as pharyngitis, scarlet fever, tonsillitis, cellulitis, impetigo, erysipelas, vulvovaginitis, pneumonia, endocarditis, meningitis, sepsis, necrotizing fasciitis and myonecrosis (1). *S. pyogenes* is one of the major causes of acute respiratory tract infections. This pathogen is known to cause autoimmune post-streptococcal sequelae, such as acute rheumatic fever and acute glomerulonephritis. Worldwide, more than 18 million people are suffering from serious GAS disease. This burden is a major cause of illness and death among children and young adults, including pregnant women, in resource poor countries (2).

Antibiotic resistance pattern of this organism has been changing in recent years and it is mainly because of inappropriate usage of broad spectrum antibiotics (3). The frequency of resistance of GAS to various antibiotics is increasing globally (4). Currently, penicillin is the drug of choice for GAS pharyngitis and penicillin resistance for GAS has not been reported yet (3, 5). However, the prevalence of antibiotic resistance among GAS is increasing day by day.

From time to time surveillance is needed to monitor the changes in antibiotic susceptibility profile of GAS in order to guide clinicians to choose appropriate antibiotics. There is not sufficient data in literature pertaining to antibiotic resistance of GAS in Indian setup till date. Therefore, this study was conducted to evaluate the prevalence and degree of antibiotic resistance among GAS.

## MATERIALS AND METHODS

A retrospective study was conducted over a period of two years from January 2016 to December 2017 in the Department of Microbiology of a tertiary care teaching hospital in southern India. All throat swabs and ear swabs from the patients having signs and symptoms of upper respiratory tract infections were included in the study. Aseptic collection of swabs was done using sterile cotton swabs and transported to the Microbiology laboratory within 2 hours. Upon arrival, sample was inoculated on 5% sheep blood agar followed by Gram staining. All cultures were incubated in 5% CO_2_ at 37°C for 24 hours. The culture plates were observed for β-hemolytic colonies. Identification of *S. pyogenes* was made based on morphology in Gram stain and beta hemolytic growth on sheep blood agar medium and bacitracin susceptibility. Identification was further also confirmed with Matrix Assisted Laser Desorption/Ionization-Time of Flight (MALDI-TOF) Mass Spectrometry (VITEK MS, bioMerieux).

Bacitracin sensitivity test was performed using 0.04 units Bacitracin discs (Himedia Laboratories, Mumbai, India) as per standard protocol. After incubation of 18–24 hours, a zone of inhibition ≥ 15 mm was considered as sensitive. Antibiotic susceptibility test was done with automated microbial identification systems, VITEK 2 (bioMerieux) and minimum inhibitory concentrations (MIC) were noted. Isolates which were resistant to two or more groups of antibiotics were considered as multi drug resistant (6). Data was analysed using SPSS 16 version.

## RESULTS

Over a span of 2 years, 2123 respiratory samples were collected, out of which 1454 were throat swabs and 669 were ear swabs. Among these, 50 specimens comprising of 42 throat swabs (84%) and 8 (16%) ear swabs were positive for *S. pyogenes.* Male predominance was seen in 27 isolates (54%). Demographic details of the patients isolates are shown in Table 1. Among 50, 30 were MDR *S. pyogenes.* Isolates resistant to two or more groups of antibiotics were considered to be MDR (7).

**Table 1.** Demographic details of cases infected by *Streptococcus pyogenes*

**Age of the patients**	**Gender of the patients**	**Throat swab (n=42)**		**Ear swab (n=8)**	

		**MDR (n=24)**	**NON MDR (n=18)**	**MDR (n=6)**	**NON MDR (n=2)**
≤18 years	Male (n=17)	10	5	2	0
	Female (n=8)	3	2	3	0
>18 years	Male (n=10)	7	1	1	1
	Female (n=15)	4	10	0	1

**Fig. 1.**
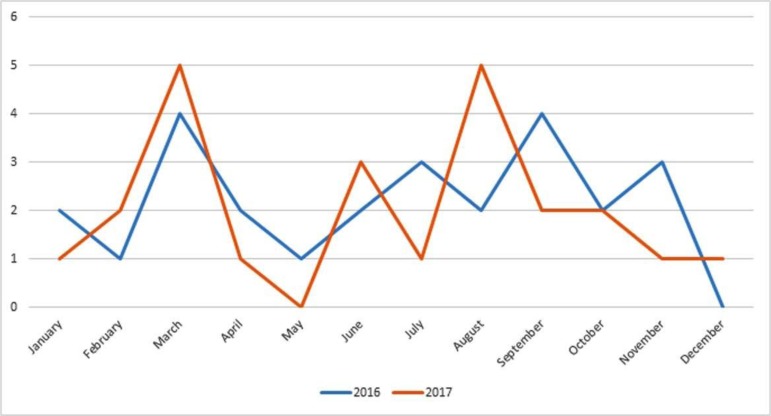
Line graph showing seasonal variation of *Streptococcus pyogenes*

Table 2 shows various other isolates obtained from throat and ear swabs. Fig. 1 shows the seasonal variation of *S. pyogenes*. The results of antibiotic susceptibility test performed by VITEK 2 are shown in Table 3. In the present study, 92.1% isolates were sensitive to penicillin and 8% were non-sensitive. Out of 50 isolates, 3 isolates were not susceptible to penicillin and their MIC range was 0.5 to 8 μg/ml. The three penicillin non-susceptible isolates are shown in Table 4.

**Table 2.** Various organisms isolated from throat swabs and ear swabs

**Name of the isolate**	**Throat swab (n=124, out of 1454)**	**Ear Swab (n=387, out of 669)**	**Number of isolates (n=511, out of 2123)**	**Percentage**
Methicillin sensitive	51	104	155	30.33
*Staphylococcus aureus*				
Methicillin resistant	21	38	59	11.54
*Staphylococcus aureus*				
*Streptococcus pneumoniae*	5	1	6	1.17
*Pseudomonas aeruginosa*	7	141	148	28.9
*Streptococcus agalactiae*	18	1	19	3.71
*Haemophilus influenzae*	5	5	10	1.95
*Acinetobacter*	3	17	20	3.91
*Escherichia coli*	6	13	19	3.71
*Klebsiella pneumoniae*	8	23	31	6.06
*Proteus mirabilis*	0	5	5	0.97
*Citrobacter koseri*	0	1	1	0.19
*Citrobacter freundii*	0	1	1	0.19
*Burkholderia cepacia*	0	2	2	0.39
*Aspergillus fumigatus*	0	5	5	0.97
*Aspergillus flavus*	0	3	3	0.58
*Aspergillus niger*	0	1	1	0.19
*Bordetella trematum*	0	1	1	0.19
*Candida species*	0	1	1	0.19
*Enterococcus faecalis*	0	3	3	0.58
*Curvularia cryoscens*	0	1	1	0.19
*Serratia marcescens*	0	5	5	0.97
*Providencia stuartii*	0	5	5	0.97
*Providencia rettgeri*	0	1	1	0.19
*Achromobacter denitrificans*	0	2	2	0.39
*Enterobacter cloacae*	0	5	5	0.97
*Enterobacter aerogenes*	0	1	1	0.19
*Morganella morganii*	0	1	1	0.19

**Table 3.** Susceptibility rates of *Streptococcus pyogenes* to different antibiotics

**Antibiotics**	**Mean Values ± SD (mm)**	**Cut off values Sensitive (CLSI)_(μg/mL)_**	**Sensitive n = (%)**	**Intermediate n = (%)**	**Resistant n = (%)**
Ampicillin (n=50)	0.76 ± 2.61	≤0.25	46 (92)	0	4 (8)
Cefotaxime (n=48)	0.40 ± 1.30	≤0.5	46 (95.8)	0	2 (4.2)
Erythromycin (n=47)	4.30 ± 3.21	≤0.25	8 (17)	0	39 (83)
Tetracycline (n=49)	7.58 ± 7.89	≤ 2	24 (49)	0	25 (51)
Levofloxacin (n=45)	2.03 ± 4.22	≤ 2	39 (86.7)	2 (4.4)	4 (8.9)
Clindamycin (n=50)	1.40 ± 0.49	≤0.25	30 (60)	0	20 (40)
Benzylpenicillin (n=38)	0.33 ± 1.31	≤0.12	35 (92.1)	3 (7.9)	0
Ceftriaxone (n=38)	0.35 ± 1.28	≤0.5	36 (94.7)	0	2 (5.3)

**Table 4.** Penicillin non-susceptible strains of *Streptococcus pyogenes*

**Strain Characteristics**	**Isolate 1**	**Isolate 2**	**Isolate 3**
Age of patients	12 years	19 years	4 years
Susceptibility to Erythromycin	Sensitive	Resistant	Resistant
Susceptibility to Clindamycin	Resistant	Sensitive	Resistant
Susceptibility to Vancomycin	Resistant	Resistant	Sensitive
Susceptibility to Tetracycline	Resistant	Sensitive	Resistant

The prevalence of cefotaxime, erythromycin, tetracycline, levofloxacin, clindamycin and ceftriaxone resistance were 4.2%, 83%, 51%, 8.9%, 40% and 5.3% respectively.

## DISCUSSION

In the present study, *Streptococcus pyogenes* showed seasonal variation. There was an increase in the number of cases during February–March and August–September in the last two years. This August–September is the monsoon season in south Karnataka which can be related to increase in number of cases of pharyngitis. During the months of February–March, there is change of weather from winter to summer, which leads to increase in number of pharyngitis cases. However, there is no documented evidence of association of pharyngitis cases with seasonal variation.

There are many therapeutic options for *Streptococcus* pharyngitis but benzathine penicillin is the drug of choice. However, clinical failures are being reported following penicillin therapy. Hence, monitoring MIC for penicillin is advisable in referral centers. In patients who are allergic to penicillin, other options such as macrolides, oral β-lactams, clindamycin or oral cephalosporins are used (7). Thus, the awareness of local antimicrobial susceptibility patterns among physicians becomes significant to select appropriate alternative treatment options.

Surprisingly, in present study 8% strains (n=3) were non-susceptible to penicillin. Their MIC was ≥0.12 μg/ml (CLSI guidelines). In this study, we found an increase in penicillin MIC ranging from 0.12 to 8 μg/ml. European Society of Clinical Microbiology and Infectious Disease reported in 2013 that MIC of penicillin was <0.25 μg/ml against Group A. Comparable results to our study with respect to the penicillin MIC were revealed from different countries: India – 0.16 to 0.75 (8), Mexico - 0.003 to 0.75 (9), Japan – 0.12 to 2 μg/ml (10). This highlights the significance of reconsidering patterns of penicillin susceptibility.

Resistance of *Streptococcus pyogenes* to macrolides is mainly due to Erm (B) or Mef (A). Erm (B) is the main indicator of high level macrolide resistance whereas Erm (A) indicates low level resistance to macrolides only. Resistance of *Streptococcus pyogenes* to macrolides ranges from 2% to 19% depending on different localities. According to Glauber et al. (11), presence of this resistance is linked to iM-LSB phenotype that is responsible for inducible clindamycin resistance. According to Muhtarova AA et al., resistance of _i_MLS_B_ phenotype was 22.55% (12). In the present study, 31.6% strains were positive for _i_MLS_B_, whereas 68.4% strains were negative. Overall clindamycin resistance was observed in 40% strains. High observed resistance can be explained as there is extensive use of macrolides for treatment of GAS resulting in increased resistance towards them. Other reason can be their easy availability over the counter. In our study, we found erythromycin MIC of 1 to 8 mg/L and resistance percentage is 83%, which is quite high when compared with other studies. Study done by Khosravi et al. (13) showed erythromycin resistance of 1%, whereas study done by Shirin et al. (14), Lu B et al. (15) and Magnussen MD et al. (16) showed erythromycin resistance of 33.9%, 94.2% and 6% respectively. Although some studies from 2002 to 2012 with no GAS resistance to ceftriaxone exist (17, 18), but 2 isolates (5.3%) of the GAS strains in the current study were observed to show higher MIC to this antibiotic, which may be due to over-usage of ceftriaxone.

In conclusion, the incidence of erythromycin and clindamycin resistance has increased. Penicillin showed good susceptibility rates but rising MIC values are alarming. In the present study, there were 3 strains with raised MIC values, which is a very significant finding as penicillin is the drug of choice for *Streptococcus pyogenes* infections. So, it is significant to reconsider the patterns of penicillin susceptibility. Clindamycin is the drug of choice for penicillin resistant GAS infections but its resistance is also on rise observed in 40% strains in the current study. This highlights the need for judicious use of antibiotics to prevent therapeutic failures.

## References

[1] Rijal KR Dhakal N Shah RC Timilsina S Mahato P Thapa S Ghimire P. Antibiotic susceptibility of Group A Streptococcus isolated from throat swab culture of school children in Pokhara, Nepal. Nepal Med Coll J 2009;11:238–240. 20635601

[2] Ralph AP Carapetis JR. Group A streptococcal diseases and their global burden. Curr Top Microbiol Immunol 2013;368:1–27. 23242849 10.1007/82_2012_280

[3] Jain A Shukla VK Tiwari V Kumar R. Antibiotic resistance pattern of group-a betahemolytic streptococci isolated from north Indian children. Indian J Med Sci 2008;62:392–396. 19008612

[4] Cizman M. The use and resistance to antibiotics in the community. Int J Antimicrob Agents 2003;21:297–307. 12672574 10.1016/s0924-8579(02)00394-1

[5] Martin JM Green M. Group A streptococcus. Semin Pediatr Infect Dis 2006;17:140–148. 16934708 10.1053/j.spid.2006.07.001

[6] Ardanuy C Domenech A Rolo D Calatayud L Tubau F Ayats J Molecular characterization of macrolide-and multidrug-resistant *Streptococcus pyogenes* isolated from adult patients in Barcelona, Spain. J Antimicrob Chemother 2010; 65: 634–643. 20118164 10.1093/jac/dkq006

[7] Choby BA. Diagnosis and treatment of streptococcal pharyngitis. Am Fam Physician 2009;79:383–390. 19275067

[8] Capoor MR Nair D Deb M Batra K Aggarwal P. Resistance to erythromycin and rising penicillin MIC in *Streptococcus pyogenes* in India. Jpn J Infect Dis 2006;59:334–336. 17060703

[9] Amábile-Cuevas CF Hermida-Escobedo C Vivar R. Comparative in vitro activity of moxifloxacin by E-test against *Streptococcus pyogenes*. Clin Infect Dis 2001; 32:S30–32. 11249826 10.1086/319373

[10] Ogawa T Terao Y Sakata H Okuni H Ninomiya K Ikebe K Epidemiological characterization of *Streptococcus pyogenes* isolated from patients with multiple onsets of pharyngitis. FEMS Microbiol Lett 2011;318:143–151. 21362024 10.1111/j.1574-6968.2011.02252.x

[11] Arêas GP Schuab RB Neves FP Barros RR. Antimicrobial susceptibility patterns, emm type distribution and genetic diversity of *Streptococcus pyogenes* recovered in Brazil. Mem Inst Oswaldo Cruz 2014;109:935–939. 25410998 10.1590/0074-0276140231 PMC4296499

[12] Muhtarova AA Gergova RT Mitov IG. Distribution of macrolide resistance mechanisms in Bulgarian clinical isolates of *Streptococcus pyogenes* during the years of 2013–2016. J Glob Antimicrob Resist 2017;10:238–242. 28735056 10.1016/j.jgar.2017.05.026

[13] Khosravi AD Ebrahimifard N Shamsizadeh A Shoja S. Isolation of *Streptococcus pyogenes* from children with pharyngitis and emm type analysis. J Chin Med Assoc 2016;79:276–280. 26874680 10.1016/j.jcma.2016.01.002

[14] Sayyahfar S Fahimzad A Naddaf A Tavassoli S. Antibiotic susceptibility evaluation of group A streptococcus isolated from children with pharyngitis. A study from Iran. Infect Chemother 2015; 47: 225–230. 26788405 10.3947/ic.2015.47.4.225 PMC4716273

[15] Lu B Fang Y Fan Y Chen X Wang J Zeng J High prevalence of macrolide-resistance and molecular characterization of *Streptococcus pyogenes* isolates circulating in China from 2009 to 2016. Front Microbiol 2017;8:1052. 28642756 10.3389/fmicb.2017.01052 PMC5463034

[16] Magnussen MD Gaini S Gislason H Kristinsson KG. Antibacterial resistance in *Streptococcus pyogenes* (GAS) from healthy carriers and tonsillitis patients and association with antibacterial sale in the Faroe Islands. APMIS 2016;124:327–332. 26833774 10.1111/apm.12513

[17] Ralph AP Carapetis JR. Group A streptococcal diseases and their global burden. Curr Top Microbiol Immunol 2013;368:1–27. 23242849 10.1007/82_2012_280

[18] Michos AG Bakoula CG Braoudaki M Koutouzi FI Roma ES Pangalis A Macrolide resistance in *Streptococcus pyogenes*: prevalence, resistance determinants, and emm types. Diagn Microbiol Infect Dis 2009;64:295–299. 19395219 10.1016/j.diagmicrobio.2009.03.004

